# Origin and Diversification of Meprin Proteases

**DOI:** 10.1371/journal.pone.0135924

**Published:** 2015-08-19

**Authors:** Ignacio Marín

**Affiliations:** Instituto de Biomedicina de Valencia, Consejo Superior de Investigaciones Científicas, (IBV-CSIC), Valencia, Spain; Laboratoire de Biologie du Développement de Villefranche-sur-Mer, FRANCE

## Abstract

Meprins are astacin metalloproteases with a characteristic, easily recognizable structure, given that they are the only proteases with both MAM and MATH domains plus a transmembrane region. So far assumed to be vertebrate-specific, it is shown here, using a combination of evolutionary and genomic analyses, that meprins originated before the urochordates/vertebrates split. In particular, three genes encoding structurally typical meprin proteins are arranged in tandem in the genome of the urochordate *Ciona intestinalis*. Phylogenetic analyses showed that the protease and MATH domains present in the meprin-like proteins encoded by the *Ciona* genes are very similar in sequence to the domains found in vertebrate meprins, which supports them having a common origin. While many vertebrates have the two canonical meprin-encoding genes orthologous to human *MEP1A* and *MEP1B* (which respectively encode for the proteins known as meprin α and meprin β), a single gene has been found so far in the genome of the chondrichthyan fish *Callorhinchus milii*, and additional meprin-encoding genes are present in some species. Particularly, a group of bony fish species have genes encoding highly divergent meprins, here named meprin-F. Genes encoding meprin-F proteins, derived from *MEP1B* genes, are abundant in some species, as the Amazon molly, *Poecilia formosa*, which has 7 of them. Finally, it is confirmed that the MATH domains of meprins are very similar to the ones in TRAF ubiquitin ligases, which suggests that meprins originated when protease and TRAF E3-encoding sequences were combined.

## Introduction

Meprins were discovered as membrane-bound proteases in rodent kidney cells [1–4] and later they were found in other vertebrates, including humans [5–9]. Mature meprin proteins have a very characteristic structure, with an N-terminal protease domain followed by a single MAM domain [10] a MATH domain (also known as TRAF domain; [6]) and a transmembrane region, which is close to the C-terminus. In human and mouse meprins, single EGF-like domains are also found, located adjacent to the transmembrane region [11–12]. The sequence of their protease domains established that meprins belong to the astacin family of metalloproteases [5, 13–15], which includes several well-known proteins, as hatching enzymes, BMP1 proteins and tolloids, among others [13, 16]. Research has generally focused on human and mouse meprins. Both species have two meprin genes, named *MEP1A* and *MEP1B* in humans (and *Mep1a* and *Mep1b* in mouse), which respectively encode the proteins known as meprin α and meprin β. Structural and functional characterization demonstrated that meprins are either bound to the cytoplasmic membrane or secreted, and that they form complexes, as dimers or multimers. More specifically, when meprin α proteins are absent, meprin β proteins generate homodimers that are anchored to the cytoplasmic membrane, although they can, in some cases, be shed and become secreted proteins. On the other hand, meprin α proteins are generally cleaved in the Golgi complex, losing their EGF and transmembrane regions, and then secreted. Once in the extracellular space, they can establish huge multimeric complexes, the largest known built by secreted proteases. In addition, heterodimers with meprin α and meprin β proteins have been found when both proteins are coexpressed in mouse cells, but apparently these heterodimers do not exist in humans (see reviews: [17–20]).

In mammals, meprins are expressed in multiple tissues, organs and cell types. They have been studied in some detail in kidney, intestine, skin and also in leukocytes and several cancer cell types [18]. However, their functions are still poorly known. They most likely have some roles in early development (see below) and they certainly have multiple functions in adults, when they may act on hundreds of different protein substrates [21]. Altered meprin expression levels have been found to contribute to renal failure in experimental models of kidney damage and also in chronic conditions which lead to cell death in the kidney (reviewed in [18, 22]). Interestingly, *MEP1A* polymorphisms have been linked to diabetic nephropathy in humans [23]. Expression in intestine and leukocytes may modulate inflammatory processes [18] and a relationship of particular allelic variants of *MEP1A* and inflammatory bowel disease has been found [24]. Also, an association of *MEP1A* variants and altered metabolism in women with polycystic ovary syndrome has been interpreted as due to an increase in the inflammatory processes related to that disease [25]. Meprins are also required in different steps of skin cell proliferation and differentiation [26] and are involved in collagen maturation [27, 28]. Some evidence for many other additional roles (modulators of angiogenesis; regulators of the number of monocytes and NK cells; in the brain, where they are involved in Amyloid Precursor Protein (APP) processing; in lungs, where they participate in the vascular remodeling processes linked to pulmonary hypertension, etc.) has been also obtained [18, 29, 30]. However, in spite of those multiple potential roles, both single and double meprin knockout mice are viable [24, 29, 31] although *Mep1b* null mice viability is lower than normal [31]. This diminished viability is the only available evidence suggesting that meprins have an early role in development.

Evolutionary studies established that meprins are a particular branch within the astacin metalloprotease superfamily [7, 8, 13, 16, 32]. It was also concluded that their origin was recent, given that they are widespread in vertebrates, but they were not detected in other animals (e. g. [7, 8, 32]). It has been shown that the MATH domain found in meprin proteins is particularly similar to those found in TRAF ubiquitin ligases, suggesting an evolutionary connection between those two protein families [33, 34]. In spite of these interesting results, the precise origin and patterns of diversification of the meprin family have never been studied in depth. All the previous evolutionary studies of meprins included only a very limited number of sequences. In this work, a detailed analysis of the evolution of meprins is developed and new facets of the patterns of diversification of this interesting group of proteins are discovered and described.

## Materials and Methods

### Sequence selection and analysis

In general, it is convenient for protein family evolutionary studies to obtain exhaustive databases, including all the available sequences in all relevant species (see e. g. [35, 36]). However, to generate an exhaustive dataset for proteases is hampered by the fact that they are extremely numerous. For example, there are between 500 and 600 protease-encoding genes in each mammalian species and similar numbers in other organisms [37, 38]. The amount of sequences to be analyzed would be in the hundreds of thousands, making subsequent phylogenetic studies very difficult. Thus, the alternative strategy of generating a broad but more specific dataset, focused on the closest relatives of meprins, was followed here. However, as it will be described in the Results section, the critical data that supported the conclusions obtained were rechecked in detail, by making exhaustive searches in all significant genomic databases, in order to establish that no relevant sequences were missed that could alter those conclusions.

First, the sequences of the three protein domains present in meprins which contain enough information as to provide a general view of the evolution of this family, i. e. the protease, MAM and MATH domains, were independently analyzed. The EGF domain was not considered, given that it is very short, rapidly evolving and also promiscuous, appearing in many different kinds of proteins. The comparisons among the results of the independent analyses of these three main domains may be used to confirm and extend the conclusions that are obtained from each one separately. Following this logic, a representative set of the proteases most similar to meprins was obtained by searching, using the BLASTP program and the whole-length human meprin protein sequences as queries, the non-redundant (nr) protein database at the National Center for Biotechnology Information (NCBI) website (http://www.ncbi.nlm.nih.gov/). After eliminating duplicates and partial sequences, a database of meprin-related proteins was obtained. These proteins were aligned using MAFFT 7.120 [39] and the alignments were manually edited using GeneDoc 2.7 [40]. All the full-length protease domains detected (1861 sequences) were selected for further analyses. Similar searches were performed using the MAM domains of human meprins as queries. In that way, and after eliminating truncated or duplicated sequences, a database containing the sequences of the 1470 MAM domains most similar to those in human meprins present in vertebrate species and two model species, the urochordate *Ciona intestinalis* and the cnidarian *Nematostella vectensis*, was obtained and the sequences were similarly aligned. Finally, the MATH domains present in the meprin-related proteases were also selected and those missing in an aligned dataset of MATH domains obtained in a previous study [34], which corresponded to recently uploaded sequences, unavailable when that study was developed, were added to it. The final alignment contained a total of 822 MATH domain sequences. The protease, MAM and MATH domain final alignments are provided as Supplementary Files (S1–S4 Tables). In addition, and in order to establish the precise evolutionary relationships among the main groups of meprins which were detected along this study, a small set of representative full-length proteins was selected, and the sequences corresponding to the three main domains, protease, MAM and MATH, were analyzed together instead of separately. By considering the three domains together, the amount of useful information is increased, thus favoring that more robust topologies are recovered in the phylogenetic trees. This global alignment is also provided as a Supplementary File (S5 Table). Finally, as indicated above, additional analyses using TBLASTN searches against the nr, htgs, gss, wgs and tsa databases at the NCBI website confirmed several of the results and conclusions obtained. These confirmatory analyses are detailed in the Results section.

### Phylogenetic and structural analyses

The evolutionary history of meprins was explored by combining phylogenetic and structural analyses. Three different methods of phylogenetic reconstruction, maximum likelihood (ML), maximum parsimony (MP) and neighbor-joining (NJ) were used in order to establish the relationships among the selected protease, MAM and/or MATH domain sequences. These analyses were performed similarly to those in several previous studies (e. g. [35, 36]). NJ and ML trees were obtained using MEGA 6.0 [41] and Maximum-parsimony (MP) trees were obtained using PAUP* 4.0, beta 10 version [42]. For NJ, Kimura´s correction was used and sites with gaps were treated using the pairwise deletion option. Parameters for MP analyses based on full-length sequences were as follows: 1) all sites included, gaps treated as unknown characters; 2) randomly generated trees used as seeds; 3) maximum number of trees saved equal to 100; and, 4) heuristic search using—depending on the particular alignment explored—either the nearest neighbor interchange (NNI) algorithm, the subtree-pruning-regrafting (SPR) algorithm or the tree-bisection-reconnection (TBR) algorithm, all three with default parameters. The particular type of search (NNI, SPR or TBR) was chosen considering the amount of sequences in each alignment, because the NNI algorithm is very fast, and thus can be used with many sequences, while the SPR algorithm and, especially, the TBR algorithm are much more expensive, and therefore they can only be used when the number of sequences is relatively small. Finally, for ML analyses, the NJ tree was taken as starting point for the iterative searches using the best model for each particular analysis, according to the ML model comparison algorithms in MEGA 6.0. The particular models are detailed in the figure legends of the corresponding dendrograms. The landscape of ML trees was always explored with the NNI heuristic. Bootstrap tests were performed to establish the reliability of the final dendrograms obtained in the NJ, MP and ML analyses. A total of 1000 replicates were generated for NJ analyses but only either 100 or, if the number of sequences was small, 200 replicates were made for the much more computer-intensive MP and ML analyses (see also details in the corresponding figure legends). MEGA 6.0 was also used to draw and edit all the trees obtained.

Whenever a single protein in our database of protease sequences was associated to a single accession number in the NCBI files, the corresponding full-length proteins were downloaded in order to characterize the domains present in them. All those proteases (a total of 1363) were analyzed using the integrated tool InterProScan 5 [43], which allows to determine their structures according to the multiple different criteria that are the basis for the most important structural databases of protein domains (such as Pfam, Smart, ProDom, etc.).

### Genomic data

Genomicus v. 79.01 (http://www.genomicus.biologie.ens.fr/genomicus-79.01/cgi-bin/search.pl) was used to characterize the synteny of *MEP1A* and *MEP1B* genes in vertebrate species. In addition, to more precisely establish the location of particular meprin genes in the *Ciona intestinalis* genome, both data at the Ensembl genome browser web page [44] (http://www.ensembl.org/) and data at the NCBI website were explored. *Ciona* operon data were obtained from the Ghost database (version 2010, updated in May 2010, and available at the web page http://ghost.zool.kyoto-u.ac.jp/). Specific TBLASTN searches at the NCBI website were performed to determine the genomic locations of the meprin-F genes of the fish *Poecilia formosa*.

## Results

**Fig 1:**
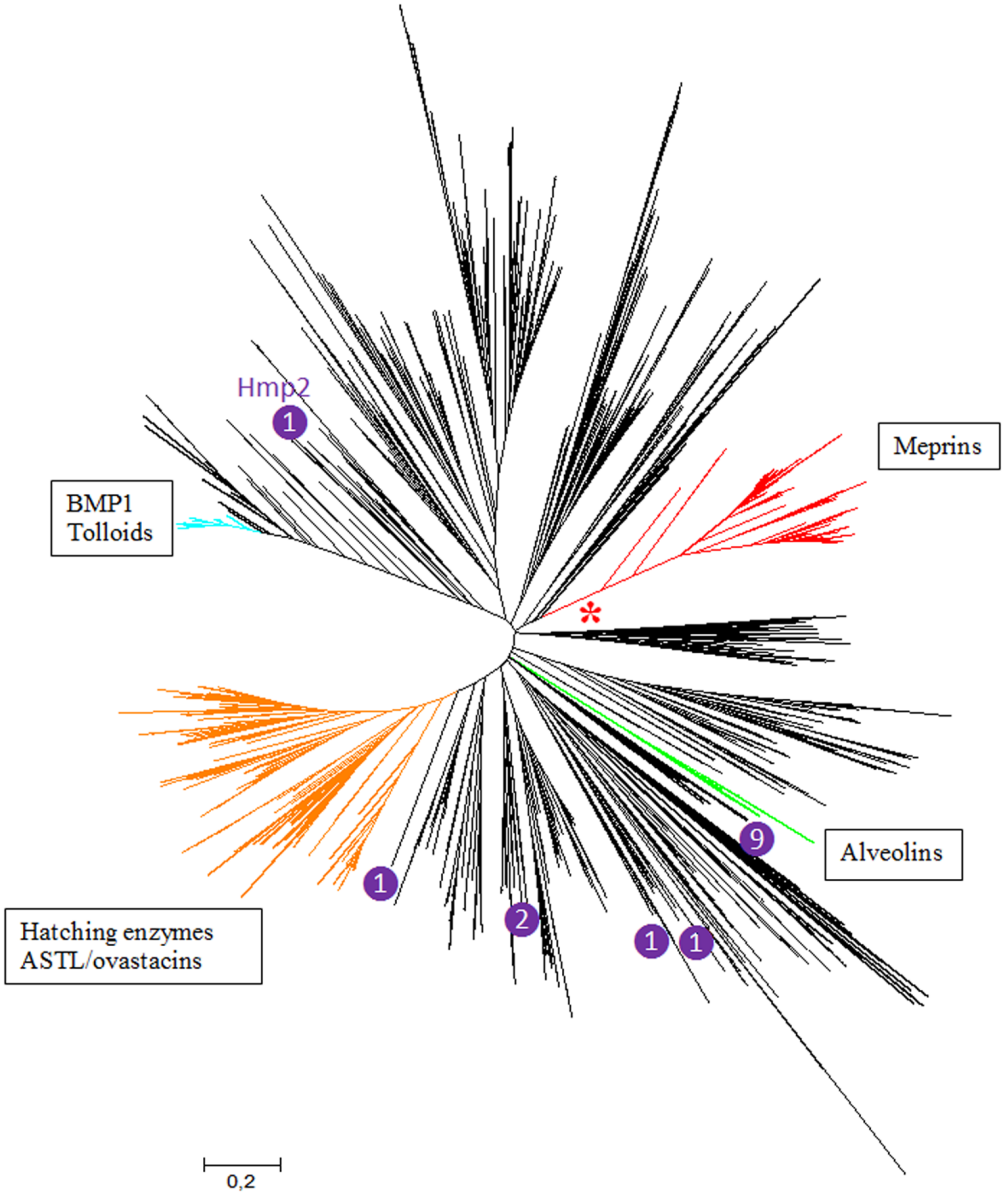
Maximum likelihood (ML) tree obtained with 1861 selected protease domains. The best model for this ML analysis was the WAG model of amino acidic substitutions with a gamma (G) distribution with five categories of sites, to account for heterogeneity in evolutionary rates (this is known as WAG + G model). The four main groups of vertebrate astacin metalloproteases are indicated (branches of colors other than black). An asterisk indicates the meprin branch (red) which is shown in detail in Fig 2. Numbers refer to how many astacins unrelated with meprin but containing MAM domains are in the respective positions in the tree. Hmp2 (top left) is a *Hydra vulgaris* protein which was proposed to be a meprin-like protease (see text), but here is shown to be just distantly related to meprins. The other MAM-containing astacins are (clockwise, starting with the largest group): nine very similar proteins found in mollusks (from the species *Aplysia californica* [3 sequences], *Todarodes pacificus* [3 sequences], *Sepiella maindroni* [1 sequence] and *Heteroligo bleekeri* [2 sequences]); two proteins of the scorpion *Tityus serrulatus*, which appear close, but, as it can be seen, slightly separated in the tree; two very similar proteins of the cephalochordate *Branchiostoma floridae*; and, finally, a single protein of the urochordate *Ciona intestinalis*.

**Fig 2:**
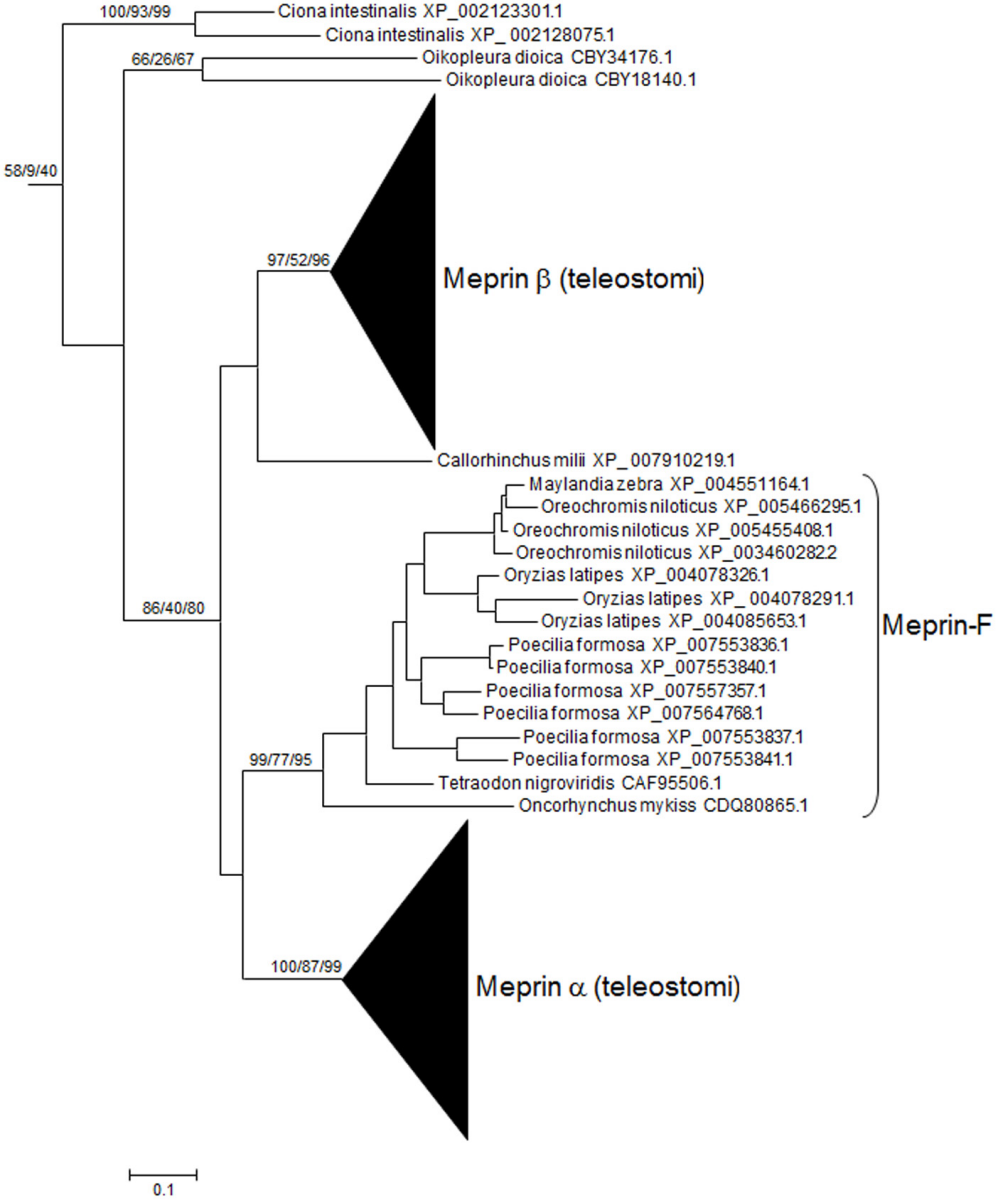
Branch of the ML tree shown in Fig 1 containing all meprins and meprin-like sequences. Numbers above the branches correspond to bootstrap values for the three methods of phylogenetic reconstruction (in the following order: NJ/MP/ML; a total of 1000 bootstrap replicates were performed for the NJ analysis, and 100 for the MP and ML analyses; the NNI heuristic search was used for MP). For simplicity, in this and the following figures, the bootstrap values are indicated only for internal, well-supported branches. Notice the presence of urochordate sequences very close to vertebrate meprins (top) and the intermediate position in the tree of some fish sequences (meprin-F; see main text).

A comprehensive database of 1861 protease domain sequences with high similarity to human meprins was obtained. Phylogenetic analyses based on these protease domains showed that all sequences obtained correspond to astacin metalloproteases [5, 7, 8, 13, 32, 45]. Particularly, all the main known vertebrate classes of astacins were detected (Fig 1). In addition to these vertebrate sequences, many sequences from invertebrates, and a few from fungi and bacteria were detected, in good agreement with previous data [16]. All known meprins appeared in well-supported branches of the phylogenetic tree (annotated as meprin α and meprin β in Fig 2). Significantly, it was observed that, in close proximity to those meprins, appeared some sequences from the urochordates *Ciona intestinalis* and *Oikopleura dioica*, albeit with low bootstrap support (see also Fig 2). The typical meprin α and meprin β sequences were detected in all types of teleostomi (i. e. bony fishes, amphibians, reptiles, birds and mammals). In most cases, a single *MEP1A* and *MEP1B* ortholog was observed, but they were duplicated in some species (as in many fishes, in the amphibian *Xenopus laevis*, etc.). This was expected, given the well-known genome duplications in some fish or amphibian lineages. In addition to these canonical meprins, there were some other sequences that appeared in intermediate positions in the tree. In particular, a single sequence from the chondrichthyan fish *Callorhinchus milii* (Acc. No. XP_007910219.1) and several sequences from bony fishes were highly divergent could not be classified in any of the two main classes, appearing in an intermediate position (Fig 2). The bony fish sequences were named meprin-F. They are abundant in some species (Fig 2), e. g. the Amazon molly (*Poecilia formosa*) has 11 meprin genes, product of small scale duplications: 2 typical *MEP1A* orthologs, 2 *MEP1B* orthologs and 7 meprin-F-encoding genes.

**Fig 3:**
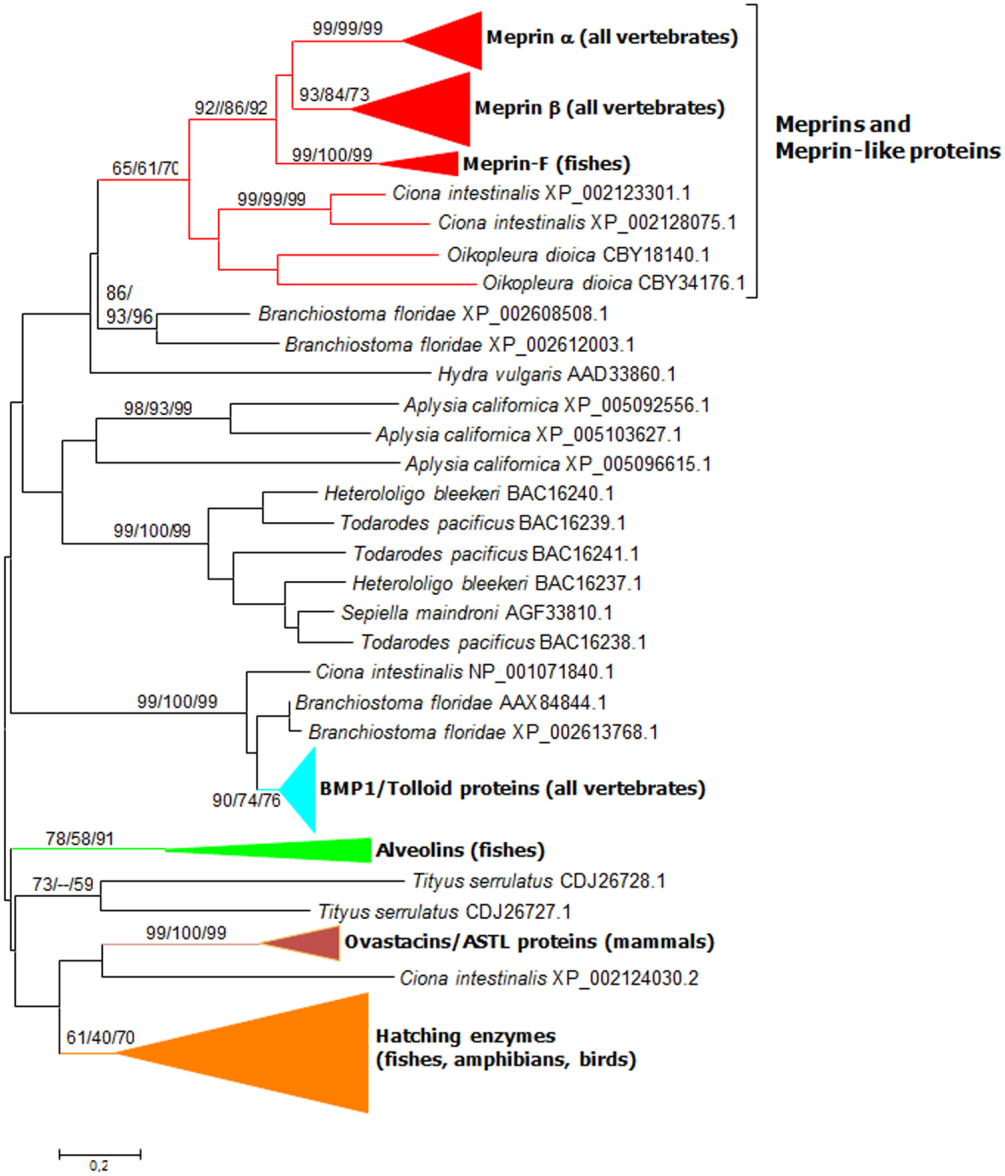
Analyses of selected protease sequences. Numbers indicate NJ/MP/ML bootstrap support, in percentages (1000 replicates for the NJ analysis; 100 for the MP and ML analyses). The ML tree was obtained using the Jones-Taylor-Thornton (JTT) with frequencies model of amino acidic substitutions, using again a G distribution and assuming some invariable sites (JTT + G + I + F model), which was selected as the most accurate in this case. MP analyses used the TBR heuristic.

An analysis of the structures of 1363 astacin proteases detected in this study allowed established that MAM domains are present in a substantial number of apparently unrelated proteins (Fig 1), indicating that these domains have been coopted several independent times by protease-encoding genes. In particular, the protease domain of the HMP2 protein of *Hydra vulgaris*, which was suggested to be a meprin [7, 45], appears, when a large number of astacin proteases are analyzed, as a distant relative of vertebrate or urochordate meprins (Fig 1). However, the mixture of highly divergent sequences used to generate Figs 1 and 2 precluded the precise characterization of the phylogenetic relationships among the meprin and meprin-like sequences. To cope with this problem, an additional analysis including only representative sequences was performed (Fig 3). As shown in that figure, the close relationships among the *Ciona* and *Oikopleura* sequences and vertebrate meprins was confirmed, while no significant bootstrap support for any connection between meprins and the rest of MAM-containing protease sequences was obtained, in perfect agreement with the general results in Figs 1 and 2. Again, an ambiguous position of Meprin-F proteins respect to the rest of vertebrate meprins was observed (Fig 3). Interestingly, the EGF domain typical of mammalian meprins was not detected in about 22% of the meprin proteins analyzed. Most of them were fish meprins, but the EGF domain was also missing in the *Ciona* meprin-like proteins detected. Previous suggestions that *Hydra* HMP2 was a meprin were based either simply on the presence of MAM domains in HMP2 [45] or on the phylogenetic analyses of very few sequences, most of them very distant relatives of meprins [6]. Once more astacin proteases are analyzed, we can safely conclude that the presence of the MAM domain in two astacin proteases does not indicate by itself a close relationship, as shown in Figs 1 and 3.

The *Ciona intestinalis* sequences shown in Figs 2 and 3 corresponded to full-length proteins and InterProScan analyses showed that they have a typical meprin structure, with protease, MAM and MATH domains and a transmembrane region. Considering that no other known proteases contain MATH domains, it was obvious that they could correspond to *bona fide* meprins. Given the interest in characterizing whether meprins were indeed present in urochordate species, a second analysis, using this time MATH domains, was performed. The logic was that if two independent analyses established that the domains present in the urochordate proteins were the closest relatives to vertebrate meprins, the hypothesis that those genes are true meprin orthologs would be strongly strengthened.

**Fig 4:**
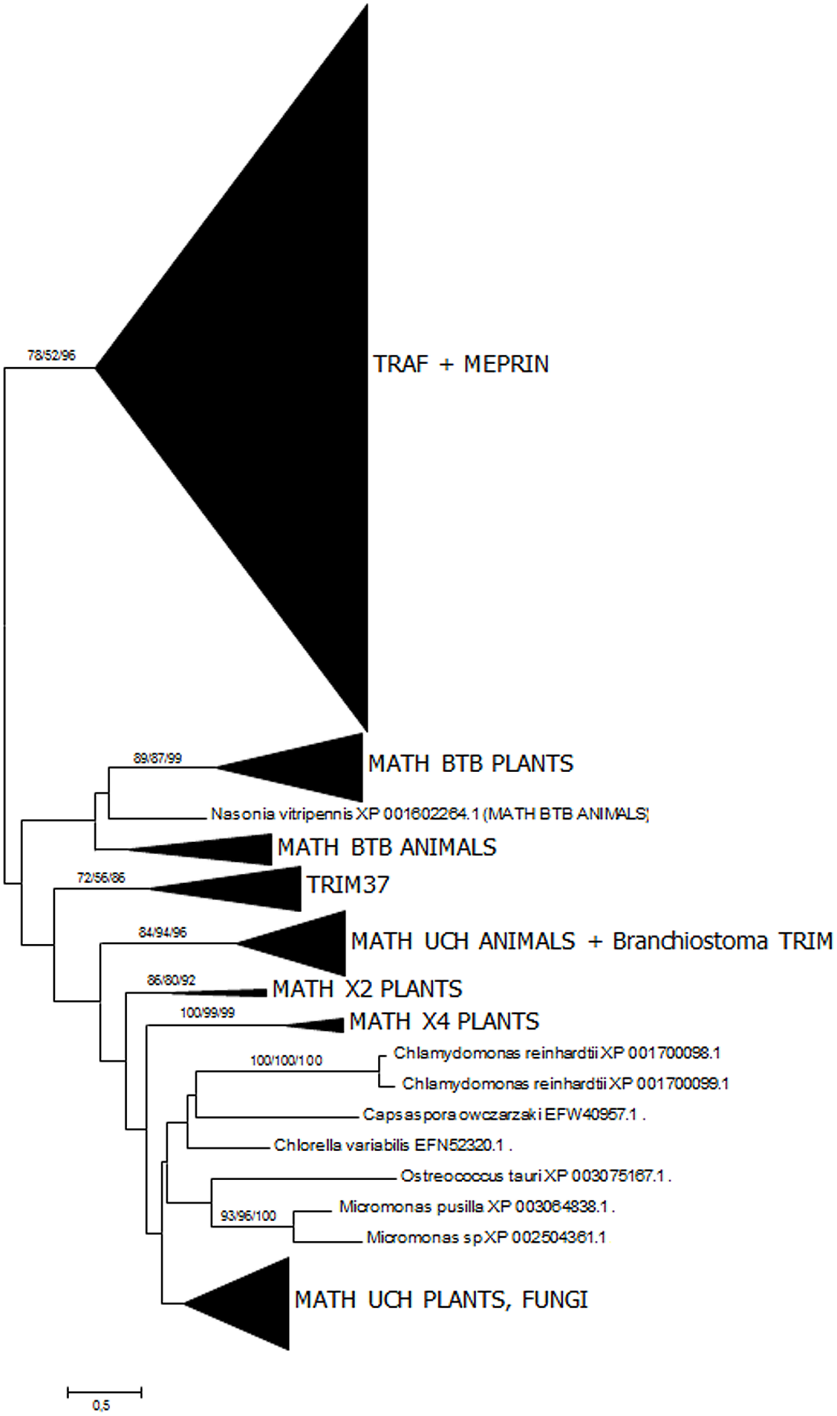
ML tree based on 822 MATH domain sequences. Numbers again refer to bootstrap support (NJ/MP/ML; 1000 bootstrap replicates for NJ, 100 for MP and ML). Meprins and TRAFs appear together in a well-supported branch. The model used in the ML analysis was the Jones-Taylor-Thornton (JTT) model of amino acidic substitutions, with G distribution (JTT+G model). MP trees were explored using the SPR algorithm.

**Fig 5:**
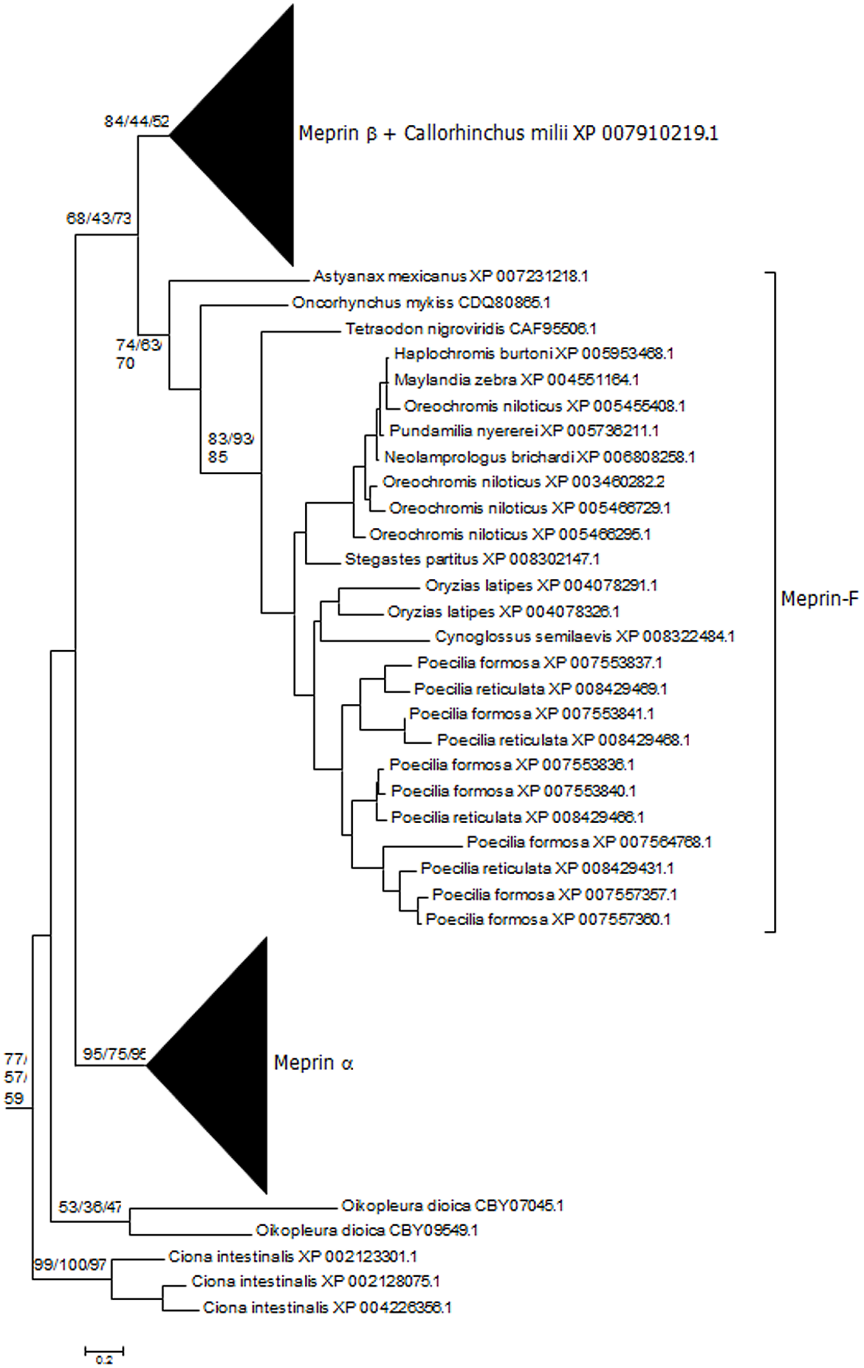
Detail of the meprin and meprin-like sequences present in the MATH ML tree. Bootstrap support as in Fig 4.

As already described in previous studies [33,34], the meprin MATH domains are extremely similar to those found in TRAF ubiquitin ligases, appearing together in the phylogenetic trees with substantially strong bootstrap support. A more extensive analysis confirming this fact is shown in Fig 4. Given that MATH domains are small and do not provide enough phylogenetic information as to determine the precise relationships among the different kinds of MATH-containing proteins ([34] and Fig 4 in this study), this similarity is striking. When the branch containing the meprin sequences was precisely examined (Fig 5), the close relationship of the typical meprin α and meprin β proteins with the urochordate sequences was again found. This is a second, independent result that confirms the protease domain-based findings, indicating that meprins exist in urochordates. The single *Callorhinchus millii* sequence mentioned above (XP_007910219.1) was also observed. However, in the MATH domain-based analyses, this sequence appeared within the meprin β group and not, as in the protease-based analyses, in an intermediate position between the meprin α and meprin β groups. Finally, meprin-F proteins were also detected. They appeared significantly closer to meprin β than to meprin α proteins (Fig 5).

**Fig 6:**
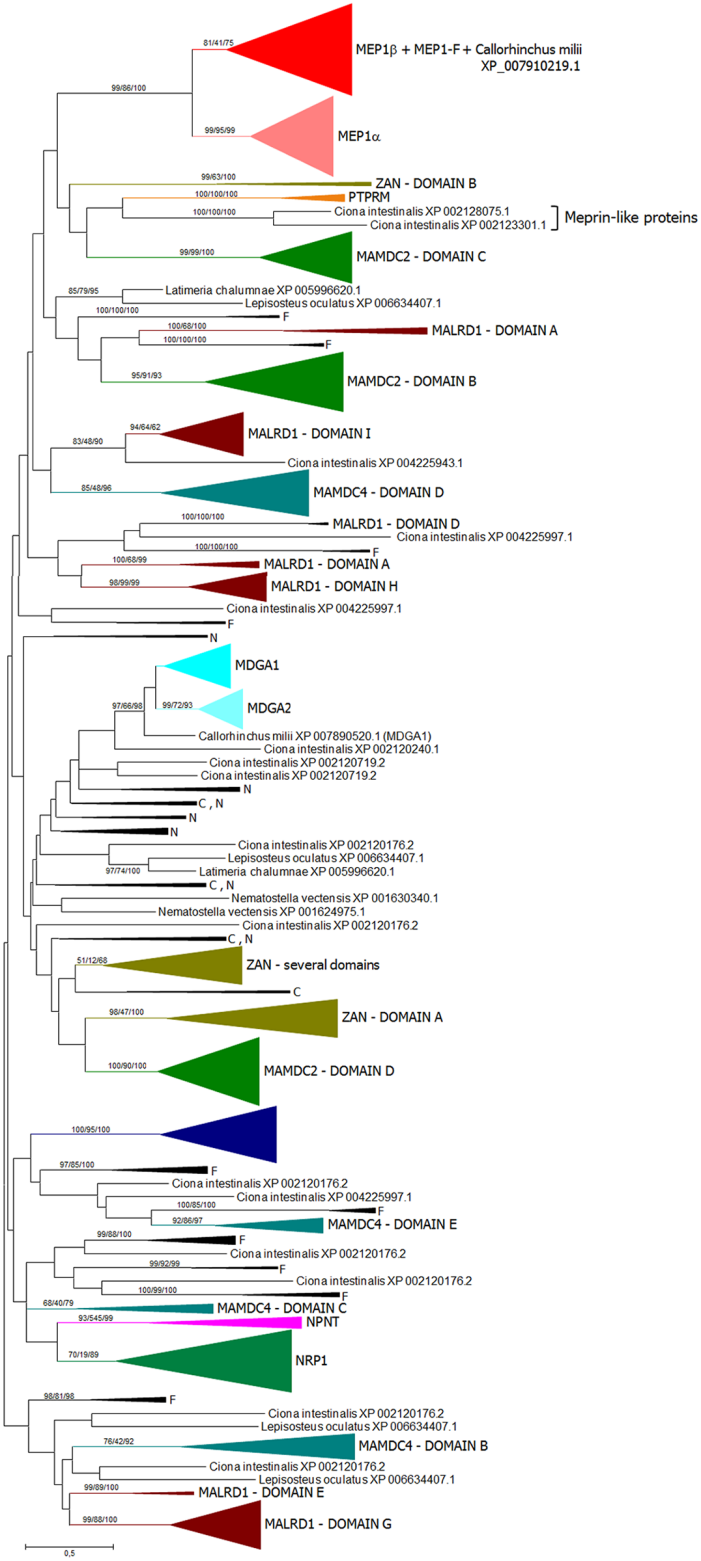
ML tree based on 1470 selected MAM domain sequences. Numbers again refer to bootstrap support (1000 replicates for the NJ analysis; 100 for MP and ML analyses). Alternative colors have been used for different proteins. When more than one MAM domain were present in a given protein, they were named with a capital letter (A, B, C etc.), assigned by moving from the N towards the C terminus of the protein. Thus, “ZAN—domain B” refers to the second MAM domain found counting from the N-terminus of the ZAN proteins. Branches labelled “C”, “F” or “N” contain sequences of, respectively, *Ciona*, *Nematostella*, or fishes. The ML analyses were performed using the JTT + G model, as in Fig 4. The MP analysis used the NNI heuristic.

To complete these analyses, a third one, this time based on the MAM domain, was performed. Given that MAM domains are present in a very large number of proteins, and some proteins contain several (sometimes many) MAM domains, it was convenient to limit the analyses to the sequences detected as most similar in BLASTP analyses to the human meprin MAM domains found in either vertebrates, the urochordate *Ciona intestinalis* or the cnidarian *Nematostella vectensis*. This last species is known to be a good outgroup to establish the early evolution of genes in animals, given its complex genome, often more similar to the ones in vertebrates than those of classical invertebrate models such as *C*. *elegans* or *D*. *melanogaster* (see e. g. [46, 47]). Finally, a total of 1470 sequences were selected and analyzed. This dataset contained MAM domains of a large variety of proteins, thus allowing a broad exploration of all the relevant sequences currently available (Fig 6). The MAM domains of all vertebrate meprins (α, β, F, and the *Callorhinchus* meprin already mentioned) appeared in two branches, with a strong support for they both being separated for the rest of branches of the tree (see bootstrap supports in Fig 6, top). Significantly, the meprin-F and *Callorhinchus* sequences appeared here within the meprin β branch. Also, the two *Ciona* MAM domains, corresponding to the proteins already found in the protease and MATH analyses, appeared quite close to meprins in the tree. However, this time they were interspersed with MAM domains found in other vertebrate proteins (encoded by the *ZAN*, *PTPRM* and *MAMDC2* genes) and did not appear as the closest relatives of meprin MAM sequences (Fig 6). This result can still be interpreted within the framework of these *Ciona* genes indeed encoding meprins, because MAM domains are short (about 150 amino acids) and also rapidly evolving compared with the protease and MATH domains. Therefore, the separation of the *Ciona* meprin MAM domains from the vertebrate meprin sequences shown in Fig 6 may be due to a rapid divergence among them or to convergence of the meprin MAM domains and those present in unrelated vertebrate proteins.

**Fig 7:**
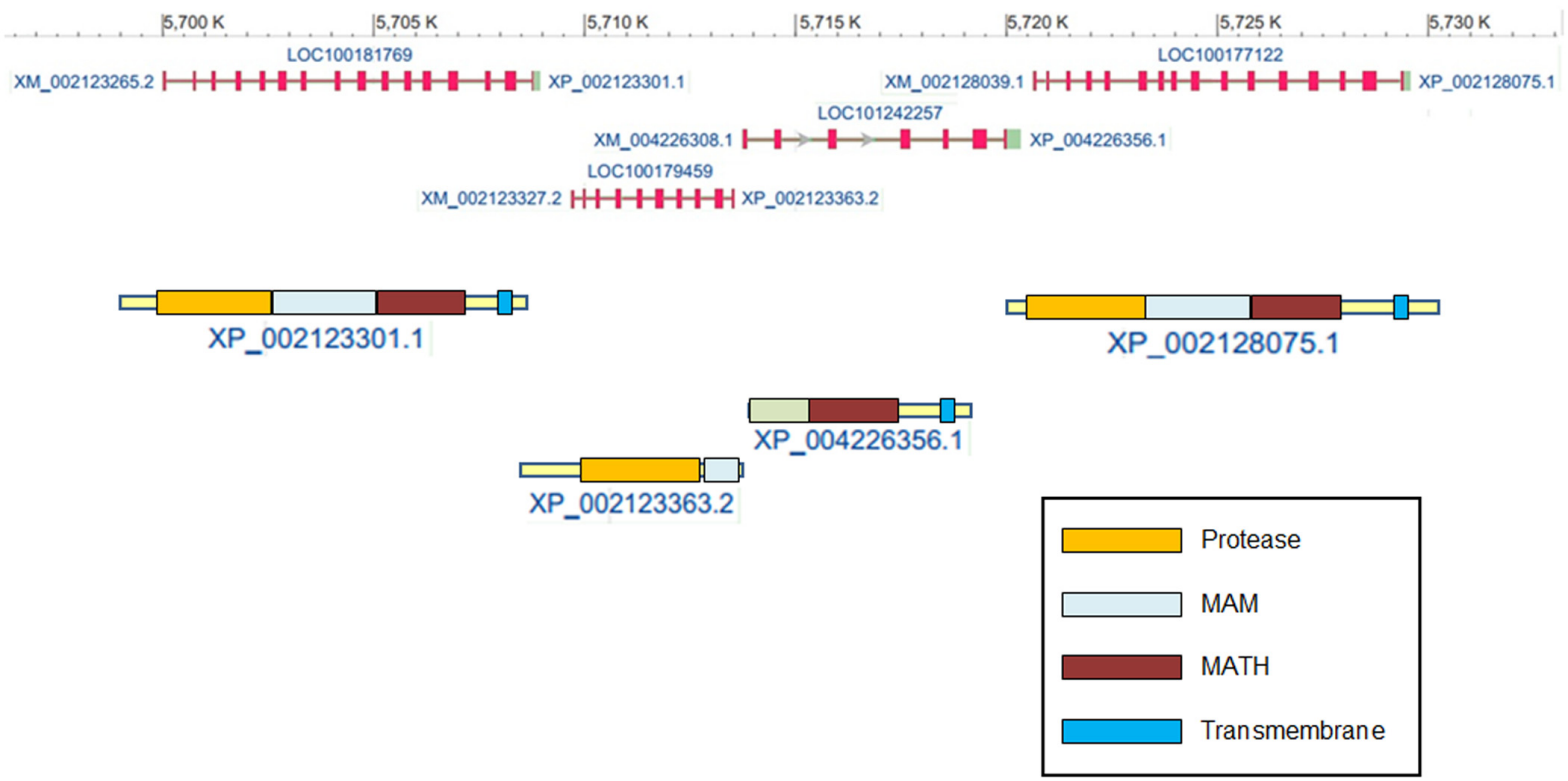
Structures of the *Ciona* meprins and locations and structures of their genes in chromosome 8 of *C*. *intestinalis*, according to NCBI data. As explained in the text, there are only three meprin genes, the two “genes” in the middle being just one, which is erroneously divided into two in the NCBI databases. Red boxes in the genes are coding exons, separated by lines corresponding to introns.

In Figs 2, 3 and 6, there are only two *Ciona* meprin-like proteins, while in Fig 5, there are three of them. This difference was explained when the genomic locations of the genes encoding these proteins were examined in detail. It was found that there are indeed three potential meprin-encoding genes in *Ciona*, located in tandem in chromosome 8 of that species (Fig 7). The discrepancy emerged because the protein NCBI databases assigned two different accession numbers to what in fact is a single protein. As shown in Fig 7, the putative genes that encode proteins with accession numbers XP_002123363.2 and XP_004226356.1 are adjacent in the genome and each one supposedly encodes half a meprin. In fact, these two putative genes appear as a single one in the *Ciona* genomic data in Ensembl (gene number: ENSCINT00000017081). This error in the NCBI protein databases indirectly led to the elimination of this protein, considered incomplete, from the protease and MAM domain analyses described above. Given their close physical proximity, it was interesting to check whether the three *Ciona* genes may be forming an operon, according to data in the *Ciona* Ghost database [48, 49], but they do not appear in the list of operons of that species.

Synteny analyses using the Genomicus genome browser indicated that both *MEP1A* and *MEP1B* genes are included in blocks of conserved genes in vertebrates. Around *MEP1A*, it has been detected a conserved cluster of genes that include *TDRD6*, *PLA2G7* and *ANKRD66*. This cluster is intact in some fish species, such as the spotted gar (*Lepisosteus oculatus*). A similar situation occurs with *MEP1B*. Although in this case conservation is less strict (both inversions and transpositions of genes to the proximity of *MEP1B* are observed), several of the genes which are close to this gene in humans (such as *TRAPPC8*, *RNF125* and *RNF138*) are also adjacent to its ortholog in many other vertebrates, including fish species. No apparent relationships were found among the genes adjacent to *MEP1A* and *MEP1B* in vertebrate species. It was also determined that at least four genes encoding meprin-F proteins in *P*. *formosa* (XP_007553836, XP_007553837 XP_007553840 and XP_007553841) are located in tandem (Acc. No. for their genomic sequences: NW_006799998.1) suggesting that selective forces may have been acting to rapidly amplify this type of genes in the fish species that carry them. For the rest of *P*. *formosa* genes, the genomic data is still too incomplete to determine whether they are tandemly arranged or not.

In order to complement the BLASTP analyses used to obtain the main datasets, additional, exhaustive TBLASTN searches against all significant NCBI nucleotide databases (see Material and methods) were now performed, in order to determine whether other potentially interesting sequences were present in them, whose corresponding proteins were not yet included in the protein databases. The emphasis was to detect additional meprin-related sequences in significant groups of animals, such as in urochordates, cephalochordates, other groups of invertebrate animals, etc. Significantly, sequences encoding fragments of putative meprins (very similar to those found in the main dataset) were detected in another chondrichthyan (*Leucoraja erinacea*; Acc. No. FL669706.1) and also in the genome of the Arctic lamprey (*Lethenteron camtschaticum*; Acc. No. APJL01063717.1). However, only a single additional fragment of a potential meprin gene was found in another urochordate, *Botryllus schlosseri* (Accession number JG362380.1), and none in cephalochordates or other non-vertebrate animals, supporting the main conclusions of this study.

**Fig 8:**
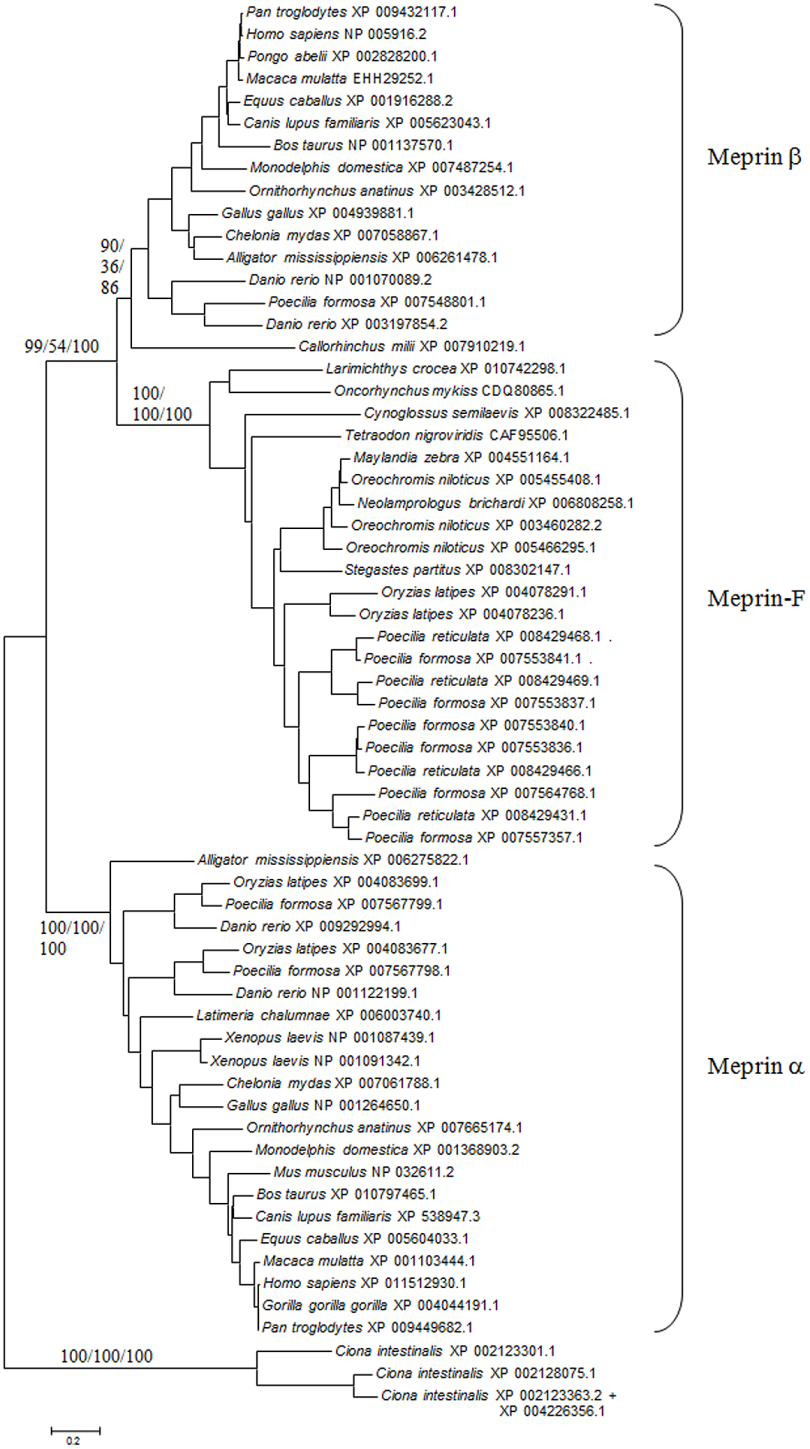
ML dendrogram showing the relationships among *bona fide* meprins, using the combined data of the protease, MAM and MATH domains. As in the previous figures, numbers refer to NJ/MP/ML bootstrap support (1000 replicates for NJ and 200 replicates for either MP or ML). Only the values for the most external, critical branches are shown. ML trees were obtained using the WAG + G + I model, while MP trees were obtained using the TBR search algorithm.

**Fig 9:**
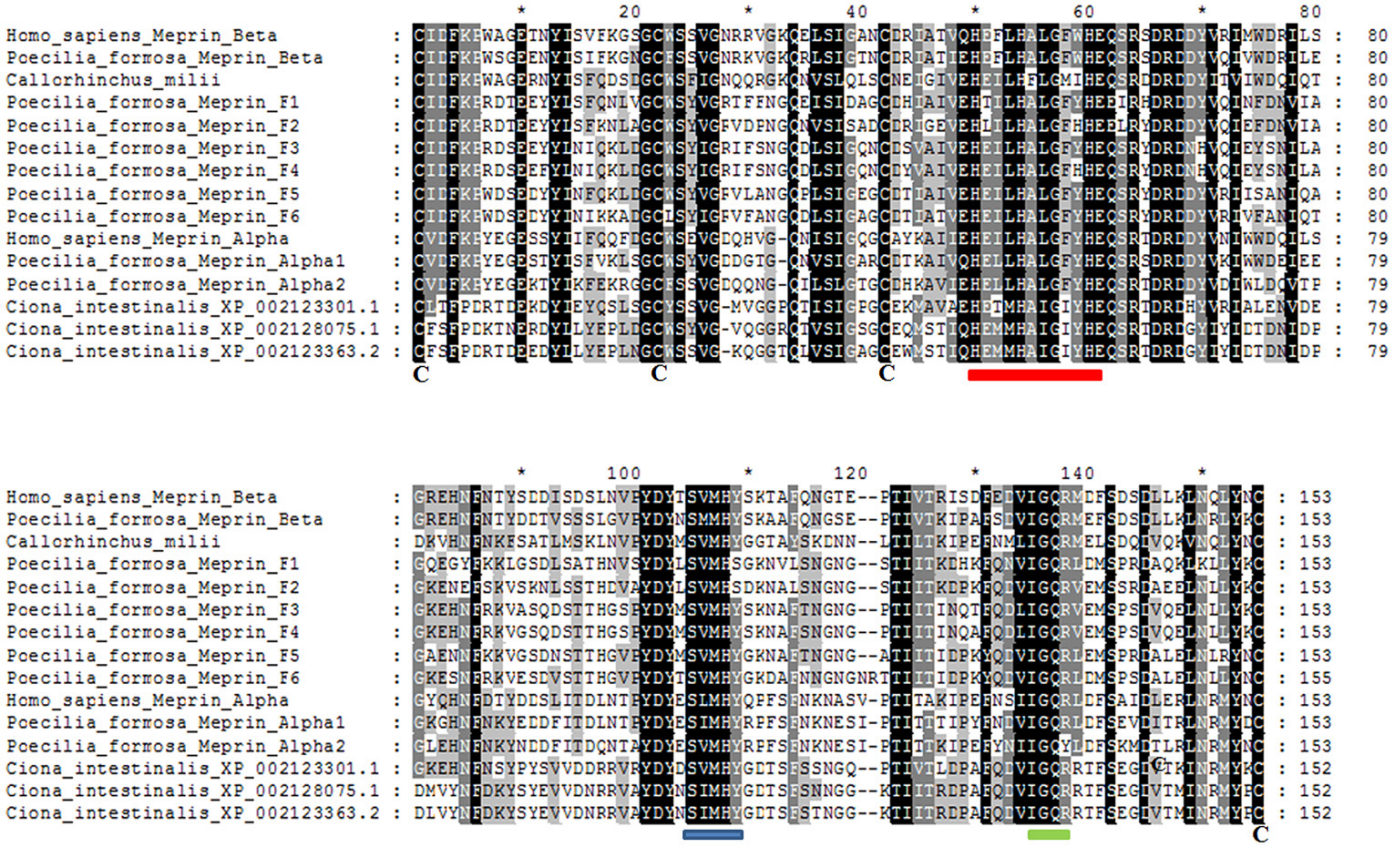
Protease domains of selected meprins. Critical cysteine residues are highlighted. Red: zinc-binding motif. Blue: Met-turn. Green: S1’ subsite [see 15 for details]. Sequences ordered as in Fig 8. Accession numbers as follows: *H*. *sapiens* Meprin β: NP_005916.2; *P*. *formosa* Meprin β: XP_007548801.1; *Callorhinchus milii*: XP_007910219.1; *P*. *formosa* Meprin F1: XP_007553841.1; *P*. *formosa* Meprin F2: XP_007553837.1; *P*. *formosa* Meprin F3: XP_007553840.1; *P*. *formosa* Meprin F4: XP_007553836.1; *P*. *formosa* Meprin F5: XP_007564768.1; *P*. *formosa* Meprin F6: XP_007557357.1; *H*. *sapiens* Meprin α: XP_011512930.1; *P*. *formosa* Meprin α1: XP_007567799.1; *P*. *formosa* Meprin α2: XP_007567798.1.

Another final point to consider was the possibility to ascertain the relationships of meprin-F and the rest of vertebrate meprins by combining all the data available for the protease, MATH and MAM domains, given that the independent analyses with each of those domains separately were somewhat ambiguous as seen in Figs 2, 3, 5 and 6. Fig 8 shows the results for the analyses using the sequences of the three domains together. The *Ciona* meprins were used as outgroups. The simplest interpretation is that meprin-F genes emerged as duplicates of *MEP1B* genes in fish species, given the substantial degree of support obtained for the meprin β + meprin-F branch (Fig 8). The *Callorhinchus milii* XP 007910219.1 sequence also appeared in this combined analysis within the meprin β group (Fig 8). Finally, Fig 9 shows an alignment of the protease domain of selected human, fish and *Ciona* meprins. As it can be easily observed, they are all very similar. The typical HEXXHXXGXXH astacin zinc-binding domain, the four cysteine residues involved in disulfide bonds as well as other characteristic motifs [15] are present in all of them. Atypical residues are however detected in a few *Poecilia* sequences, a fact that may be related to their functional differentiation. Particularly, *Poecilia* Meprin_F1 and Meprin_F2 (Fig 9) show a serine residue in a position of the Met-turn in which a tyrosine, involved also in zinc binding, is generally found in all astacins ([13]; corresponds to position 109 in Fig 9). Also *Poecilia* Meprin_Alpha2 shows a substitution of a characteristic arginine by tyrosine in the S1’ subsite (position 138 in Fig 9). This arginine is known to be involved in the preference of meprins to cleave at acidic residues [50].

## Discussion

Meprins are typical astacin proteases, but with a peculiar structure that allows to explore their evolution quite simply, by considering together structural changes (the acquisition of new protein domains) and sequence similarity. Here, it has been shown that proteins with typical meprin structures and sequences are encoded in the genome of urochordates. In particular, three genes are present in tandem in the *Ciona intestinalis* genome (Fig 6). It is significant that the *Ciona* sequences were missed in a previous study that specifically studied that species [8], probably because the *C*. *intestinalis* genome was incompletely sequenced at that time. These results and the lack of meprins in cephalochordates indicate a precise time of origin for meprin proteins, before the urochordate/vertebrate split, which is earlier that so far assumed. Of course, more complex hypotheses can be proposed, involving an even earlier emergence plus subsequent losses in some lineages, such as cephalochordates or invertebrates, but those hypotheses would be less parsimonious, given the available data.

Another interesting finding is the fact that some fish species have a significant number of additional meprin genes encoding quite divergent proteins, here called meprin-F (Figs 2, 3, 5, 6 and 8). The combined results for the protease, MATH and MAM domains indicate that meprin-F genes are *MEP1B* duplicates (Fig 8). The finding of meprin sequences in chondrichthyans and one jawless fish (see previous section) are also significant. The combined analyses suggest that the chondrichthyan sequences are related to *MEP1B* (Fig 8). An interesting point that remains to be determined when more data from these early-branching fishes become available is whether they indeed contain a single meprin gene, as so far observed, or they actually have two. This will allow to determine when the *MEP1A/MEP1B* duplication occurred.

This study also contributes to a better understanding of how meprin proteases acquired their characteristic domains. We have seen that independent acquisitions of MAM domains by astacin proteases have occurred quite often (Fig 1), probably allowing or at least facilitating protease dimerization [51]. This same conclusion was reached in a related study, albeit those analyses included only a limited number of sequences [52]. These multiple cooptions suggest that meprins originated from an ancestor already containing a protease and a MAM domain, with the MATH domain being coopted just once, precisely in the progenitor of urochordates and vertebrates, leading to the emergence of this gene family. The close similarity of the MATH domains of TRAF ubiquitin ligases and meprins ([33, 34] and this study), together with the fact that TRAF genes are widespread in metazoa [33], thus being older than meprin genes, indicate that the MATH domains of these proteases derived from sequences originated in a TRAF-encoding gene.

A final point to consider is the new views of meprin function that these results may provide. Understanding the roles of the *Ciona* proteins may contribute to new insights into meprin fundamental functions and a better comprehension of which functions of the vertebrate meprins are ancient and which ones evolved more recently. Also, the finding that some meprins, and particularly those found in *Ciona*, lack the typical EGF domain found in mammalian proteins, may provide significant clues about the potential roles of these domains in the meprins of different species.

## Supporting Information

S1 Table Fasta alignment (txt file) corresponding to Fig 1. (FASTA)

S2 Table Fasta alignment (txt file) corresponding to Fig 3. (FASTA)

S3 Table Fasta alignment (txt file) corresponding to Fig 4. (FASTA)

S4 Table Fasta alignment (txt file) corresponding to Fig 6. (FASTA)

S5 Table Fasta alignment (txt file) corresponding to Fig 8. (FASTA)
